# Symptom appraisal and help- seeking before a cancer diagnosis during pregnancy: a qualitative study

**DOI:** 10.3399/BJGP.2024.0208

**Published:** 2025-04-08

**Authors:** Afrodita Marcu, Emma Ream, Karen Poole, Jo Armes, Faith Gibson, Lisa Whittaker, Jenny Harris

**Affiliations:** School of Health Sciences, University of Surrey, Guildford.; School of Health Sciences, University of Surrey, Guildford.; Clinical Trials and Statistics Unit, The Institute of Cancer Research, Sutton.; School of Health Sciences, University of Surrey, Guildford.; School of Health Sciences, University of Surrey, Guildford; director of research, Nursing and Allied Health, Centre for Outcomes and Experience Research in Children’s Health, Illness and Disability, Great Ormond Street Hospital for Children NHS Foundation Trust, London.; Blood Cancer UK, London.; School of Health Sciences, University of Surrey, Guildford.

**Keywords:** cancer, early diagnosis, early presentation, gestational cancer, pregnancy, qualitative research

## Abstract

**Background:**

The estimated incidence of a cancer diagnosis during or shortly after pregnancy is 1 in 1000 pregnancies in England. Pregnancy can have an impact on symptom appraisal and help-seeking for symptoms subsequently diagnosed as cancer. Little is known about the pathway to cancer diagnosis in pregnancy or delays that women can encounter.

**Aim:**

To explore symptom appraisal, help-seeking decisions, and experience of receiving a cancer diagnosis during pregnancy.

**Design and setting:**

Semi-structured interviews were conducted with women diagnosed with cancer during or shortly after pregnancy in the previous 4 years in the UK, recruited between January and May 2022 via the charity Mummy’s Star.

**Method:**

This study used reflexive thematic analysis of 20 interviews. Analysis was largely inductive and the themes generated were mapped onto the intervals of the Model of Pathways to Treatment.

**Results:**

Symptoms were often interpreted through the lens of pregnancy by both participants and most of the healthcare professionals from whom they sought help. Participants who found breast lumps were likely to suspect cancer and be referred promptly for tests in secondary care. Although most participants sought timely help for their symptoms, some subsequently encountered health system delays, partly owing to both the vague nature of their symptoms and the COVID-19 pandemic.

**Conclusion:**

Health services need to better support women presenting with possible cancer symptoms during pregnancy to ensure timely diagnosis. Recommendations include prioritising symptoms over attributing them solely to pregnancy, ensuring timely referrals to rule out serious conditions, and emphasising clear communication alongside robust safety-netting practices. A full assessment is essential before dismissing symptoms as pregnancy related.

## Introduction

Cancer in pregnancy has an incidence rate of about 1 in 1000 deliveries in England, with breast, melanoma, cervical, haematological, ovarian, and colorectal cancers being the most frequently diagnosed.^1^ Women diagnosed with cancer during pregnancy or postpartum have a lower 5-year survival compared with those diagnosed during periods remote from pregnancy.^2^ Timely diagnosis is critical because it can have an impact on women’s prognosis, treatment options, and treatment outcomes,^3^^,^^4^ as well as decisions around continuation of pregnancy.^5^ There is, however, emerging evidence that pregnancy has an impact on how women interpret their symptoms and seek help.^5^^,^^6^ Common presenting symptoms such as breast lumps, nipple or vaginal discharge, fatigue, abdominal pain, nausea, or rectal bleeding can be attributed to pregnancy, resulting in delayed presentation or referral to secondary care.^3^^,^^7^ Difficulties in cancer diagnosis during pregnancy may vary by cancer symptom signature, that is, nature and frequency of symptoms with which women present.^8^ Cancers with broader symptom signatures (for example, colorectal and blood cancers) may be more difficult to diagnose during pregnancy than cancers with narrower symptom signatures (for example, breast cancer, melanoma, and thyroid cancer) that can manifest through easily recognisable symptoms, for example, lumps.^8^

Delays in cancer diagnosis can arise at different stages of the diagnostic pathway, from the symptom appraisal interval, that is, time from detecting symptoms to deciding they warrant medical attention, to the help-seeking interval, that is, time from perceiving a reason to discuss symptoms with a healthcare professional (HCP) to first consultation, and the diagnostic interval, that is, time from first presentation to an HCP to formal diagnosis, as detailed by the Model of Pathways to Treatment (MPT, Supplementary Figure S1).^9^ Evidence suggests that pregnant women with breast cancer experience extended symptom appraisal intervals compared with matched (non-pregnant) controls (median 35 versus 23 days).^10^ Further, they can encounter diagnostic delays for breast cancer of 2–18 months compared with non-pregnant women because symptoms can be masked by the physiological changes of pregnancy.^7^^,^^10^ Consequently, more pregnant women are diagnosed with advanced breast cancer than non-pregnant women (65%–90% versus 45%–65%).^7^

Limited research exists on women’s experiences of cancer diagnosis during pregnancy,^11^ particularly regarding delays in presentation and/or diagnosis. In the UK, to our knowledge, only one qualitative study has explored women’s emotional experience of a cancer diagnosis in pregnancy;^12^ it did not attempt to understand women’s experiences of the journey to diagnosis and focused exclusively on three women with breast cancer. Our research addressed this gap and used the MPT framework to map symptom appraisal, help-seeking, and the diagnostic process to understand factors affecting timely diagnosis.

**Table:** How this fits in

There is a gap in current understanding about the experiences of women diagnosed with cancer during or around pregnancy including how they appraise and seek help for cancer-related symptoms. This qualitative study found that women and healthcare professionals often interpreted symptoms through the lens of pregnancy, particularly when symptoms were vague. Health professionals need to ensure full assessment of symptoms, timely referral, and effective safety-netting for these women.

## Method

### Recruitment

Women with a diagnosis of cancer in pregnancy in the UK were recruited to participate in online interviews (January to May 2022) via Mummy’s Star, a UK organisation supporting women and families facing cancer during, or within a year of, pregnancy. Owing to the relative rarity of a cancer diagnosis during pregnancy, we used a convenience sample of users of Mummy’s Star’s online forum, who responded to our study advert placed on the forum. Recruitment was open to those with any type of cancer to ensure the study recruited women with experience of a wide range of cancers and experiences of the pathway to a cancer diagnosis.

Prior to interview we did not screen whether women had experienced prolonged pathways in their diagnostic journey. Eligible women were ≥3 months postpartum and had been diagnosed with cancer during or up to 3 months following pregnancy, irrespective of pregnancy outcome (for example, live birth, termination, miscarriage, or stillbirth). We excluded women diagnosed with cancer ≥4 years before the study was conducted. Following advice from our patient and public involvement representatives and Mummy’s Star support workers, we avoided recruiting women who were pregnant or had given birth in the previous 3 months to minimise the potential for distress.

### Interviews

Semi-structured online interviews (see interview schedule, Supplementary Information S1) explored women’s experience of receiving a cancer diagnosis during pregnancy. The interview schedule was informed by the MPT, past research on symptom appraisal and help-seeking,^13^ and input from our patient and public involvement representatives. Participants first shared their diagnosis experience uninterrupted. Then, the interviewer probed symptom interpretation, help-seeking strategies, and the diagnostic process. As typical with semi-structured interviews, the researcher was free to follow up and probe emerging issues specific to each woman’s experience. The participants were also asked to provide, verbally, demographic information and details about the type of cancer they had been diagnosed with. The interviewer was a mother of a child born just before the COVID-19 pandemic, and this helped establish rapport^14^ with participants. Participants were compensated £25.

### Patient and public involvement

Two public members, previously diagnosed with cancer during pregnancy, acted as patient and public involvement representatives. They guided study design, recruitment, and materials, aiding data interpretation to ensure our findings authentically mirrored the cancer diagnosis experience during pregnancy. The patient and public involvement representatives met virtually with the research team and were also consulted via email. They advised on: the questions to be included in interview schedule; phrasing of questions to minimise distress; the optimum time in the pregnancy and cancer journeys to recruit and interview participants; the interpretation of the data; and the mapping of the findings onto the MPT.

### Analysis

Interviews were anonymised, transcribed verbatim, and analysed using reflexive thematic analysis,^15^^,^^16^ whereby themes generated through analysis are *‘creative and interpretive stories about the data, produced at the intersection of the researcher’s theoretical assumptions, their analytic resources and skill, and the data themselves’*.^15^ We followed Braun and Clarke’s six phases of data engagement, coding, and theme generation:

- data familiarisation and writing familiarisation notes;
- systematic data coding (coding is open and codes represent units of meaning);
- generating initial themes from coded and collated data;
- developing and reviewing themes;
- refining, defining, and naming themes; and
- writing the report.^16^

Themes represented patterns of shared meaning around a central concept. The analysis was inductive and iterative as each interview was read multiple times, and the codes and themes were revisited to enhance clarity, cohesiveness, and rigour. The first author conducted interviews and led data analysis. Three other authors independently read and coded a selection of transcripts (the second, fourth, and senior author). Preliminary themes were refined collaboratively, then deductively mapped onto the MPT. NVivo R1 software supported analysis. As the concept of data saturation is less applicable to reflexive thematic analysis,^17^ our sampling was guided instead by the concept of ‘information power’,^18^ which is typically determined in a qualitative study by the: study aim, sample specificity, use of established theory, quality of dialogue, and analysis strategy.

Our study had information power in the sense that: it had a narrow research question; it had sample specificity, that is, participants held characteristics specific to the study aims; it gathered rich in-depth data and the quality of the dialogue between interviewer and participants was strong; and the data were analysed in depth and with support from established theory, that is, the MPT.

## Results

### Sample characteristics

Twenty-six women responded to the advert. Two were ineligible because of a current pregnancy, and four did not complete consent forms or confirm interview dates. The final sample comprised 20 women, aged 27–45 years (median 35.5) (Table 1 and Supplementary Table S1). Fourteen (70%) had been diagnosed during COVID-19, the remainder before it.

**Table 1.** Characteristics of the participants (*N* = 20)

**Characteristic**	**Participants, *n* (%)**
**Cancer type**	
Breast cancer	13 (65)
Bowel cancer	2 (10)
Hodgkin lymphoma	2 (10)
Non-Hodgkin lymphoma	1 (5)
Thyroid cancer	1 (5)
Melanoma	1 (5)

**Stage at diagnosis**	
1	1 (5)
2	3 (15)
3	4 (20)
4	4 (20)
Not remembered/indicated by the participant	8 (40)

**Parity**	
First child	9 (45)
Second child	10 (50)
Third child	1 (5)

**Ethnicity**	
White British	15 (75)
Scottish	3 (15)
Mixed ethnic background	2 (10)

**Marital status**	
Married	10 (50)
Cohabiting	7 (35)
Engaged	2 (10)
Getting divorced	1 (5)

**Educational attainment**	
Postgraduate diploma/Masters	4 (20)
Degree	8 (40)
A-levels	4 (20)
Foundation degree	1 (5)
College	1 (5)
Level 4 diploma	1 (5)
NVQ level 3	1 (5)

**Geographical spread**	
London	5 (25)
West Sussex	3 (15)
Yorkshire	2 (10)
Other counties of England	7 (35)
Scotland	3 (15)

In total, 17 of the 20 (85%) were diagnosed during pregnancy (5–35 weeks), and three (15%) postpartum (6–16 weeks), having initially sought help for symptoms during pregnancy. Half gave birth full term (>37 weeks); the rest had pre-term births (33–36 weeks) following induction or caesarean section. Breast cancer was most common (*n* = 13/20, 65%), including various subtypes (Supplementary Table S1). Two women had recurrent (metastatic) disease following initial diagnosis pre-pregnancy.

### Themes

The interviews lasted 36–66 min. The analysis generated nine themes that we mapped onto the MPT intervals to detail the pathway to a cancer diagnosis during pregnancy (Box 1). One help-seeking interval theme was specific to breast cancer symptoms (provided in Supplementary Information S2). Additional quotes for each theme are shown in Supplementary Box S1.

**Box 1.** Mapping the themes onto three intervals of the Model of Pathways to Treatment

**Symptom appraisal interval**	**Help-seeking interval**	**Diagnosis interval**
• Interpreting symptoms through the lens of pregnancy	• Symptoms incongruent with pregnancy	• HCPs attributed symptoms to pregnancy or other health conditions
• Suspecting cancer before formal diagnosis	• Initial symptoms getting worse	• Re-presenting in primary care before referral for cancer tests
	• Concerned about breast changes (Supplementary Information S2)	• HCPs made referrals for cancer tests
		• Appraisal of the timeliness of diagnosis

*HCP = healthcare professional.*

### Symptom appraisal interval

#### Interpreting symptoms through the lens of pregnancy

Many participants made sense of their symptoms through a pregnancy lens, informed by familiarity with bodily changes that are considered ‘normal’ during pregnancy, for example, abdominal pain:

*‘Because it was low down in my colon, it felt like it was womb-related* […] [I thought] *it must be something to do with the baby, because it was so low, something to do with the pregnancy. I never thought, “It could be something in my colon.”’*

(Participant [P]10, bowel cancer)

Some participants looked up their symptoms online and were falsely reassured that they were pregnancy related, partly because they added ‘pregnancy’ to their searches.

Participants’ symptom interpretation did not end after their first consultation with an HCP but continued up to diagnosis (which in some cases involved multiple presentations to HCPs). Beliefs that symptoms were pregnancy related were sometimes reinforced by HCPs’ initial assessments or their lack of evident concern:

*‘It wasn’t until I was pregnant and then the lump got a lot thicker and I developed quite a large lump very quickly. I don’t know whether it was because of COVID, but, because I was pregnant, I was just put under “it’s changes because of pregnancy” and they didn’t even want to see me at that point. Which obviously to me it made sense, you are pregnant, your breasts change. They weren’t concerned. Obviously, they are the professionals. I just listened to what their advice was.’*

(P11, breast cancer)

A few participants put symptoms down to pre-existing pregnancy-related health conditions (for example, symphysis pubis dysfunction and previous miscarriage).

Some participants suspected that their symptoms might be cancer but wanted to believe they were pregnancy related. Hesitance to attribute symptoms to cancer despite having ‘red flag’ symptoms was mostly reported by those with breast cancer:

*‘Of course* [cancer] *crossed my mind because it was a lump in my breast, but because I was pregnant I was just using that as a hook, well, I’m pregnant, it’s not going to be breast cancer. Trying to rationalise it, I felt it was much likely because I was pregnant, to be something pregnancy or hormone related. So maybe I just told myself that.’*

(P4, breast cancer)

#### Suspecting cancer before formal diagnosis

Many participants reported a ‘gut feeling’ of concern over their symptoms and suspected cancer:

*‘I’ve always checked myself because I knew I was quite vulnerable to any sort of skin cancer. I think I’ve always been aware of the risks and what the potential could be, but this was different.* [Mole] *looked different to anything I’d dealt with before and so I knew it wasn’t right, I just didn’t know in what respect.’*

(P12, malignant melanoma)

Others suspected their symptoms might be cancer after looking them up online:

*‘I put “lump on neck pregnancy” into Google and it had come up with an article of a woman who had Hodgkin’s lymphoma when she was pregnant. So that was already in my mind before I went, I was already frightened.’*

(P1, Hodgkin lymphoma)

However, not all were influenced by information gathered online; some dismissed cancer as a possible explanation for their symptoms despite cancer appearing in search results:

*‘When I was reading online, I didn’t think it could be cancer. I mean, it did come up when I was doing my research, but I thought I don’t think I’ve had any of those symptoms like the night sweats. I didn’t feel like any of it applied to me, apart from obviously the swelling was the concerning thing.’*

(P19, non-Hodgkin lymphoma)

### Help-seeking interval

Some participants mentioned their symptoms to their midwife, while others sought help directly from their GP. Reasons for not mentioning symptoms to midwives included lack of time, lack of direct contact during the COVID-19 pandemic, seeing different midwives at different appointments, or *‘not thinking it was going to be anything serious’* (P19, non-Hodgkin lymphoma). Participants were encouraged by midwives to see their GP after disclosing symptoms; in one case the midwife referred the participant to a direct access service (where HCPs can refer directly to specialist diagnostic and treatment services) for further investigation of a breast lump.

### Symptoms incongruent with pregnancy

Motivations to seek help for symptoms varied. For example, two women diagnosed with bowel cancer had different symptoms that in turn influenced motivation. Participant 15 had blood in her stool, saw this as incongruent with pregnancy, and contacted her GP promptly:

*‘I just started getting blood in my stools and I knew something wasn’t quite right. And I did some research about pregnancy and as far as I could gather, you can have bleeding but it tends to be much later on when you are very heavily pregnant and you can get haemorrhoids. But because I was so early, I didn’t think it fit, so I contacted the local GP.’*

(P15, bowel cancer)

The unusual nature of symptoms at particular timepoints in pregnancy made some participants seek prompt help from their GP:

*‘I knew how much breast changes there were and lumps and bumps, milk ducts and everything, all that was not unusual but I did think, gosh, approximately eight weeks, that feels odd. So I wasn’t unduly concerned, I was thinking it was something hormonal to do with being pregnant, but I made an appointment with the doctor for the next week.’*

(P4, breast cancer)

#### Initial symptoms getting worse

After monitoring symptoms, several participants contacted their GP or visited healthcare settings such as accident and emergency or maternity wards because of severe pain or worsening of symptoms:

*‘When I was about three months pregnant, I noticed a really small lump in my breast. I didn’t think much of it, I kept an eye on it. And I was getting stabbing pains as well. After about two months, I noticed that it hadn’t gone away and it had actually got bigger. So I went to my GP who then referred me to the breast care clinic.’*

(P20, breast cancer)

### The diagnostic interval

#### HCPs attributed symptoms to pregnancy or other health conditions

Participants’ accounts of HCPs reactions to their symptoms — and their decisions to refer them or not for clinical investigations — revealed that some HCPs had attributed symptoms to pregnancy, reassuring participants that these were ‘normal’ or ‘hormonal’:

*‘I mentioned the symptoms to the midwife and I also phoned the GP because my back was very sore, but they thought the baby was lying on a nerve and this was why I was getting shooting pains up the arm and up my back.’*

(P8, Hodgkin lymphoma)

Some, who consulted multiple times, reported being made to feel *‘silly’* (P8), *‘overly anxious’* (P8), or *‘hysterical’* (P16) first-time mothers. For example, Participant 8 who was subsequently diagnosed with stage 4 bulky Hodgkin lymphoma:

*‘One doctor suggested I was just overly anxious because I’m a new mum and it made me feel silly because I’m thinking I’m not making it up but if they could see me in the actual surgery, they could see that there were lumps there, it wasn’t in my head.’*

(P8, Hodgkin lymphoma)

Among some participants, with cancers other than breast, symptoms were ascribed to other health conditions despite symptoms persisting and/or worsening:

*‘So initially I thought I had a really bad ear infection, so I went to my local GP, he had a look at my ear and he said, “Yes, it’s an ear infection”, and they prescribed me whatever it was to spray my ear. I noticed after about a week it was making no difference, so I went back to my GP and they re-prescribed it, had a look in my ear, said, “Yes, the ear canal is red. It just looks generally sore.”’*

(P19, non-Hodgkin lymphoma)

Even when participants presented with symptoms typical of breast cancer, for example, bleeding from the nipple, some HCPs were reported as attributing these to pregnancy or other causes. However, it is not possible to know whether these HCPs suspected cancer or were simply trying to be reassuring:

*‘My nipple was bleeding on a daily basis, and when I had my follow-up appointment on the phone and I spoke to a different consultant, by that point I was about thirteen weeks pregnant, they said because my breasts were changing maybe it was taking longer to heal, so probably nothing to worry about but if the bleeding persisted to get back in touch with them.’*

(P2, breast cancer)

#### Re-presenting in primary care before referral for cancer tests

Some participants, particularly those with cancers other than breast, presented multiple times at their GP or in antenatal settings as their symptoms persisted, or had various tests in secondary care before being referred for cancer tests:

*‘I went* […] *three times — this would have been in November, maybe once in October and twice in November* [2018] *— and said to them, “I am really worried about this pain that I am having”* […] *I think I went three times, and they did the same thing every time. They didn’t dismiss me, but I guess it was my first pregnancy, and I was just worried about the baby.’*

(P10, bowel cancer)

Lack of face-to-face appointments during the COVID-19 pandemic made some participants contact their GPs repeatedly and/or chase up their referrals to secondary care. This suggests that in some cases the COVID-19 pandemic affected the usual cancer care pathway and associated safety netting for diagnostic uncertainty:

*‘At that point, I had given up. I was like, it can’t be anything serious. So, I didn’t chase it up again until September twenty-twenty, my mole started to bleed, which I knew wasn’t normal. I was really concerned at that point, so I went back to my GP, “This is the fourth time I’ve contacted you. No one’s seen me face-to-face and I know this isn’t normal. Can you please do something?”’*

(P12, malignant melanoma)

#### HCPs made referrals for cancer tests

Women who sought help for common symptoms such as breast lumps seemed to have timely referrals. Among participants later diagnosed with breast cancer, GPs reassured them that symptoms might be pregnancy related but promptly referred them for secondary care tests:

*‘The GP didn’t seem unduly concerned … with hindsight, she may have just been putting on a calm front, but I felt reassured. The GP did an exam and said, “I’m pretty confident it is just because you are pregnant; however, the breast clinics are well funded, you’ll get in and seen quickly, so let’s just be on the safe side and refer you.” I was nine weeks pregnant.’*

(P4, breast cancer)

#### Appraisal of timeliness to diagnosis

Many participants described in intricate detail the time it took to receive their cancer diagnosis, noting the weeks or months to diagnosis and treatment. Some blamed lack of face-to-face appointments during the COVID-19 pandemic for any delays in diagnosis, for example, Participants 8 and 11 who experienced lumps but were only diagnosed 3–4 months postpartum:

*‘I waited a few weeks* [after seeing the ear, nose, and throat specialist] *and then I had to go back for an ultrasound to biopsy the neck, and it was only in December that I got told it was Hodgkin’s* [*sic*] *lymphoma. I was told* […] *it was spread around the body and it had entered the lung, so I got told all this just prior to Christmas but had* [daughter] *in September and obviously three months prior to that was when I started complaining about the symptoms.’*

(P8, Hodgkin lymphoma)

One participant submitted a formal complaint to the NHS about delays encountered in referral and diagnosis — the response confirmed that she had met the criteria for urgent referral but that HCPs had not adhered to practice guidelines. Others were angry with themselves for not chasing referrals or asking to be seen sooner.

Some participants thought the delays in diagnosis were owing to the misattribution of symptoms to pregnancy, as in the cases of Participants 11 and 18 who were eventually diagnosed postpartum with stage 3 breast cancer despite presenting with breast lumps during pregnancy. Similarly, Participant 10’s bowel cancer was stage 4 by the time she was diagnosed:

*‘By the time they took the tumour out in January, and then they scanned me in February, we found out that it had gone to my liver.* [pause, participant upset] *Whether it was already going to go to my liver or not, you don’t really know. But if I had been diagnosed earlier in the pregnancy, definitely it would have helped with my situation, life expectancy.’*

(P10, bowel cancer)

Paradoxically, some participants felt lucky not to have been diagnosed earlier in pregnancy as this would have marred their enjoyment of it or led to difficult choices around continuation of pregnancy. They argued that *‘ignorance was bliss’* (P8) and the delay in diagnosis was *‘a blessing in disguise’* (P16) as it allowed *‘a bubble of joy and happiness’* (P8):

*‘In hindsight maybe I’m glad I didn’t know I had breast cancer right at the very beginning of the pregnancy because I think if I had it would’ve clouded the whole pregnancy.’*

(P2, breast cancer)

## Discussion

### Summary

This is, to our knowledge, the first comprehensive qualitative study in the UK to both explore women’s recent cancer diagnosis experiences during pregnancy, drawing on other cancers beside breast,^12^ and consider these in relation to the MPT^9^ to describe this pathway. It addresses the dearth of understanding around the challenges to timely diagnosis and initiation of treatment that pregnant women can experience.^11^ It reveals how cancer symptoms can be (mis)interpreted by women and HCPs, and in some patients lead to prolonged intervals to diagnosis. Pregnancy serves as a heuristic^19^ in women’s and HCPs’ interpretation and decisions to act on the symptoms, with referrals to cancer care typically occurring for red flag symptoms or after exhausting other treatments. Women with breast cancer who were aware of, and sought help for, definitive symptoms of the disease often had timely referrals. However, some of those with cancers featuring broader symptom signatures,^8^ or non-lump breast symptoms, reported experiencing diagnostic delays because of symptom normalisation and pregnancy-related misattribution. Vague, non-specific symptoms, particularly those associated with blood cancers, are known to hinder timely cancer diagnosis,^20^ and their misattribution can be exacerbated by pregnancy, thereby potentially prolonging diagnosis further.

Pregnancy, besides serving as a heuristic, introduced an additional layer to patient–HCP interactions resembling diagnostic overshadowing (misattribution of symptoms of one illness to an already diagnosed comorbidity).^21^ Some HCPs were reported to have dismissed concerns and repeated presentations as signs of first-time mothers’ anxiety.

Some participants expressed frustration over slow diagnosis and delays influenced by diagnostic overshadowing^21^ and remote consultations. Previous research on timeliness of cancer diagnosis^22^ has highlighted its impact on patient satisfaction. Paradoxically, some participants found benefits from delayed diagnosis: allowing more time to enjoy pregnancy and avoiding potential terminations or cancer treatment,^12^ highlighting the complex emotions at the intersection of cancer and pregnancy.

### Strengths and limitations

The diversity of the sample in terms of cancer type, symptom signature, and public awareness strengthened the study. This facilitated a broad picture of how different cancer symptoms are appraised during pregnancy by both women and HCPs, highlighting potential misattributions to pregnancy. Typically, studies applying the MPT focus on one cancer^23^ but because of the relative rarity of pregnancy-related cancer this was not feasible or appropriate.

There were some limitations to our study. We did not recruit participants from a clinical setting, where we could have purposively screened them based on clinical characteristics; instead they were from forum users of a cancer charity. It is possible that they are not representative of the population of women diagnosed with cancer in pregnancy in the UK. This was a pragmatic decision as retrospective cohort studies suggest that cancer in pregnancy is inconsistently recorded in electronic patient records,^24^ which can make purposive recruitment in clinical settings challenging. Our sample also included women who were diagnosed before or during the COVID-19 pandemic; therefore, it is difficult to distinguish pandemic-related delays from other factors affecting cancer diagnosis in pregnancy. Although 70% (*n* = 14/20) of our participants were diagnosed during the COVID-19 pandemic, there is little evidence in our study that the pandemic influenced their symptom appraisal or intentions to seek help. The pandemic only appeared to affect the diagnostic interval in a few cases, although a further limitation is the extent to which this can be objectively established from the participants’ narratives and distinguished from other factors contributing to health services delays. Furthermore, as we recruited participants from among the forum users of a charity, our findings may be more indicative of the experiences of women who chose to access support than of those who do not.

Finally, as this was a qualitative interview study, we cannot draw firm conclusions as to whether the prolonged diagnostic pathway experienced by some women necessarily led to late-stage diagnosis. However, a key strength of our approach was the involvement of three experts by experience (two PPI representatives and one co-author who was previously a support worker at Mummy’s Star) who not only provided guidance on recruitment, methods, and study conduct, but also provided critical feedback on our emergent findings.^14^

### Comparison with existing literature

This is, to our knowledge, the most comprehensive qualitative study in the UK to explore women’s experiences of cancer diagnosis in pregnancy (with the previous study including only three women)^12^ and to focus specifically on women’s experience of receiving a cancer diagnosis.^11^ Like past research applying the MPT to understand pathways to cancer diagnoses and potential sources of delay, the MPT helped map symptom experience, help-seeking, and diagnostic processes.^25^ However, the utility of applying the MPT in the context of cancer during pregnancy was somewhat hindered in participants with multiple presentations in primary care. Symptom appraisal and help-seeking strategies did not strictly follow linear paths, as the interpretation of symptoms, by both women and HCPs, continued beyond the initial presentation until formal cancer diagnosis. This aligns with past observations that the MPT, although visualised as linear, should recognise that people can have multiple primary care visits and adjust their symptom appraisal based on clinical encounters, moving back and forth between intervals.^26^

### Implications for research and practice

A summary of our initial recommendations for clinicians is provided in Box 2. This study reveals how pregnant women and HCPs (mis) interpret cancer symptoms during pregnancy, highlighting potential delays in diagnosis. HCPs’ management of diagnostic uncertainty and resulting diagnostic delays can distress patients, highlighting the need for future research on awareness raising and practice recommendations. Diagnosing cancer associated with pregnancy is challenging because of symptom complexity and vague presentation often attributed to normal pregnancy. Improving services to promptly diagnose potential cancer symptoms in pregnancy is crucial, necessitating education for primary care, midwifery, and obstetrics/gynaecology to recognise and thoroughly assess symptoms before dismissing them as pregnancy related.

**Box 2.** Initial recommendations for midwives and GPs

**Relevant findings and evidence**	**Recommendation**
**Midwives**	
Our results suggest that HCPs attributed symptoms to pregnancy or other health conditions. Not all cancer symptoms will be usual ‘red flags’, but, even when they are, people were sometimes reassured without full assessment	**See the symptom not the pregnancy** Some cancer symptoms may be masked by pregnancy It is important to be aware that some common experiences in pregnancy may also occur in cancer Listen to women, explore their ‘gut feelings’
Women will be seeing midwives regularly as part of routine antenatal appointments and our results suggest that they will talk to their GP about a symptom if their midwife recommends it. Because appointments are time limited and women will not always see the same midwife, it is important to make a clear record of the recommendation to see the GP so it can be followed up at the next antenatal appointment	**Refer and rule it out** If a woman presents with a symptom that you are concerned could be cancer, speak to the GP on call and ask them to see the patient. Where available, you may be able to refer to direct access clinics If in doubt, always ask the woman to book a follow-up appointment with the GP Make a note in the electronic health records/handheld maternity notes to check that when you (or one of your midwifery colleagues) next see the woman, you check that she attended the GP appointment
**GPs**	
Our results suggest that women often interpreted their symptoms through the lens of pregnancy and may have deferred help seeking. Women can be diagnosed with a range of cancers during pregnancy,^25^ and HCPs including GPs sometimes attribute symptoms to pregnancy or other health conditions	**Assess the symptom not the pregnancy** Women can by diagnosed with a range of cancers during pregnancy Assess their symptoms as you would do in a non-pregnant woman including physical examination, usual baseline tests, and clinical history Women may put off seeking help during pregnancy — check how long they have had the symptoms and if they have spoken to other HCPs about their symptoms
GPs were vital for making referrals for cancer tests and this route can avoid women having to present via emergency routes	**Referral and communication** If you are concerned it could be cancer, make a prompt referral, explain why you are making the referral, and try to avoid giving false reassurances
Although for most women their symptoms will not be cancer, it is important to safety net as our evidence suggests women re-present multiple times in primary care before referral for cancer tests and some women only access care via emergency routes	**Safety netting** If you suspect symptoms are not cancer, agree a management plan with the woman and tell them how long you would expect the symptoms to last Give the woman a specific date to return if the symptoms do not ease, and let them know what to do if they worsen Reassure them that returning is welcome and encouraged

*HCP = healthcare practitioner.*

## Data

The research data are not shared because they contain sensitive and potentially identifying information.
